# Special Correspondence

**Published:** 1884-05

**Authors:** 


### Special Correspondence.

THE PROPOSITION TO INCORPORATE A SCHOOL OF MIDWIFERY IN THE STATE OF NEW YORK.

MESSRS. EDITORS: There are now two bills to be presented to the N. Y. Legislature at Albany, one of which is to incorporate the "New York Maternity (Hospital) and school of Midwifery," and the other is an "Act to Regulate the Practice of Midwifery in the State of New York." The last gives midwifery a legal status—allowing women who have a certificate of six months' course of study the right to practice midwifery in the State of New York. These bills were brought to the attention of the Medical Society of the County of New York in February by Dr. A. W. Warden, who was immediately appointed one of a committee to report on the matter. He kindly furnishes us with the facts here given. His minority report, which was adopted by a large majority of the society after a lively discussion at their meeting of March 24, is as follows:

"Medical Society of the county of New York, S. O. Vander-Poel, M.D., President; W. M. Carpenter, M.D., Secretary.

"The undersigned, a part of your committee appointed to investigate and report to the Medical Society of the County of New York the result of investigations relative to two acts proposed for State legislation, presented to the Medical Society of the county of New York for approval by the promoters of the proposed 'New York Maternity and School of Midwifery,' reports as follows:

"First, in regard to the proposed New York Maternity and School of Midwifery, your committee recommend that the Medical Society of the County of New York do not endorse or commend the incorporation of such an institution, as not conducive to the elevation of the medical profession and the good of the community.

"Secondly, in regard to the proposed 'Act in relation to the practice of midwifery in the State of New York,' your committee find such proposed law to be not advisable, and counsel the Medical Society of the County of New York, in the interest of maintaining a high standard of qualification for the practice of midwifery, and of all branches of the profession of medicine, and in the interest of the people of the State of New York, *not to approve* any State legislation giving the right to practice midwifery, or any branch of the profession of medicine, to persons other than qualified and registered (male and female) physicians."

(Signed,) DR. A. W. WARDEN.

The *New York Times*, March 25, giving a report of the meeting at which this minority report was adopted, says:

"The ground taken by the doctor, opposed to the licensing of midwives, was that this would tend to lower the standard of the medical profession. The practice is in vogue on the Continent and in England, and has proved anything but satisfactory. It was claimed that if adopted, it would result in many ignorant persons getting license, after a few months' study, and that the number of deaths among newly-born children would increase largely. The action of the County Medical Society has been looked for with great interest by the doctors throughout the country, as there are similar movements on foot in Baltimore and Chicago. A number of the members of the County Medical Society will go to Albany to oppose the bills before the legislative committees."

---

THE VIRUS OF HYDROPHOBIA.—M. Pasteur made an interesting communication to the Paris Academy of Sciences, on February 26, in relation to canine madness. He stated that the disease could be communicated to a dog by inoculation with fragments of marrow or of nerve taken from a mad-dog. He also stated that he had rendered twenty dogs proof against the disease by inoculating them with a modified virus,

